# Glycemia affects glomerular filtration rate in people with type 2 diabetes

**DOI:** 10.1186/s12882-019-1584-7

**Published:** 2019-10-29

**Authors:** E. Jennifer Weil, Sayuko Kobes, Lois I. Jones, Robert L. Hanson

**Affiliations:** 1 Phoenix Epidemiology and Clinical Research Branch, Phoenix, AZ USA; 2 Present Address: Division of Renal Medicine, Emory University School of Medicine, Atlanta, GA USA; 3 National Institute of Diabetes and Digestive and Kidney Diseases, 1550 E Indian School Rd, Phoenix, AZ 85014 USA

**Keywords:** Type 2 diabetes mellitus, Estimated glomerular filtration rate, Hyperfiltration, Glycemic variables, Kidney function

## Abstract

**Background:**

In type 2 diabetes (T2DM), the Chronic Kidney Disease Epidemiology Collaboration (CKD-EPI) equation for estimated glomerular filtration rate (eGFR) systematically underestimates the measured adjusted glomerular filtration rate (aGFR) when aGFR is high. We studied the extent to which glycemic variables associate with kidney function, and developed equations including these variables that estimate aGFR in people with T2DM.

**Methods:**

Diabetic Pima people had aGFR measured from iothalamate clearance divided by body surface area. eGFRs < 60 ml/min/1.73m^2^ were excluded. Multivariate linear regression identified variables correlated with kidney function. We constructed equations for approximating aGFR. Correlation analysis and 10-fold cross-validation were used to compare the CKD-EPI equation and the new approximating equations to the measured aGFR. Ability to detect hyperfiltration, defined as aGFR > 120 ml/min/1.73m^2^, was compared by analysis of receiver-operating (ROC) curves.

**Results:**

aGFR was measured 2798 times in 269 individuals. HbA1c, fasting plasma glucose (FPG), age, and serum creatinine (SCR) were significantly associated with aGFR. The best equations for approximating aGFR used HbA1c and FPG in addition to age and SCR. They approximate aGFR in this cohort of obese people with T2DM more precisely than the CKD-EPI equation. Analysis of ROC curves show that these equations detect hyperfiltration better than does the CKD-EPI equation.

**Conclusions:**

HbA_1c,_ FPG, age, and SCR yielded the best equations for estimating aGFR in these subjects. The new equations identify hyperfiltration better than the CKD-EPI equation in this cohort and may inform clinical decisions regarding hyperfiltration in individuals with T2DM.

## Background

Glomerular filtration rate (GFR) measures an important aspect of kidney function. Measurement of GFR requires infusion of inulin, iothalamate, iohexol, ^51^Cr-EDTA, gadolinium-DTPA or gadolinium-DOTA [1]. The adjusted GFR (aGFR) is the measured GFR divided by body surface area (derived from height and weight) and indexed to 1.73m^2^.

Many organizations recommend use of *estimated* aGFR (eGFR) using the Chronic Kidney Disease Epidemiology Collaboration (CKD-EPI) equation [2], which is based on sex, race, age and serum creatinine (SCR), in clinical practice [3–5]. Since kidney disease caused by T2DM is the most frequent cause of kidney disease in the United States [6, 7], clinicians often estimate aGFR using the CKD-EPI equation to make healthcare decisions in people with diabetes.

The CKD-EPI equation generally tends to underestimate aGFR, and the higher the aGFR the more severe the extent of underestimation [8]; this underestimation is often attributed to differences between the populations in which the CKD-EPI equation was derived and those in which it has been subsequently applied. Due to this inaccuracy at high aGFRs, when the eGFR is equal to or greater than 60 ml/min/1.73m^2^, clinical labs often report results as “≥60 ml/min/1.73m^2^”. An additional potential limitation of the CKD-EPI equation for estimating of aGFR in individuals with diabetes is that it does not consider measures of glucose which can drive aGFR up [9–12]. This inaccuracy in identifying hyperfiltration is a potential limitation of the CKD-EPI equation [13]. Although there is little direct evidence that restraint of hyperfiltration decreases incidence of diabetic kidney disease [14] some studies are suggestive that this is the case [15–22]. Thus, individuals with hyperfiltration might be candidates for renoprotective therapy.

The purpose of the present study was to determine the extent to which glycemic variables, along with other standard clinical variables, account for the difference between the CKD-EPI eGFR and measured aGFR in diabetic Pima people, a Native American population with a high prevalence of type 2 diabetes [23, 24]. The other purpose of the study was to develop equations that more closely model aGFR to identify hyperfiltration in individuals in this cohort.

## Methods

### Study subjects and design

From 1965 to 2007, the Pima from Arizona participated in a longitudinal study of diabetes. All individuals age ≥ 5 years were invited to have a biennial health examination, which included a 75 g oral glucose tolerance test for diagnosis of diabetes. In addition, adults (ages 18–65) with type 2 diabetes from this population were invited to participate in studies that included repeated measurements of GFR in the context of several studies of the natural history of diabetic kidney disease and one that tested the renoprotective efficacy of losartan in early diabetic nephropathy (ClinicalTrials.gov number, NCT00340678) [25–28]. All GFR studies were performed between 1988 and 2014.

### Clinical and anthropometric measures

Fasting plasma glucose was measured on several autoanalyzers over the years 1988–2014 using a hexokinase method (Boerhinger Mannheim/HK). HbA1c was measured by high performance liquid chromatography (HPLC). Serum specimens for measurement of creatinine concentration were stored at − 80 °C until the assay which was performed within 30 days of sample collection. SCR was measured by a modified Jaffé reaction until 2011 [29], and by an enzymatic method thereafter. Samples were calibrated initially to the laboratory at the Cleveland Clinic and later became traceable to an isotope-dilution mass spectrometry measurement procedure. Glomerular filtration rate (GFR) was measured in the morning after an overnight fast by infusion of iothalamate. An HPLC system was used to measure iothalamate concentrations. Serum and urinary measurements were made in four different collection periods and an average of these was taken to get the GFR [30]. Average urinary clearance of iothalamate was equated with the GFR. Height was measured at the first examination for each protocol and weight was measured at each measurement of GFR. aGFR was calculated as measured GFR divided by BSA, calculated using the Du Bois equation [31].

eGFR (in ml/min/1.73m^2^) was calculated according to the CKD-EPI formulae for non-black race with SCR measured in mg/dl [2]:

$$ {\displaystyle \begin{array}{ll}\mathrm{eGFR}=144\times {\left(\mathrm{SCR}/0.7\right)}^{\hbox{-} 0.329}\times {(0.993)}^{\mathrm{age}}& \mathrm{if}\ \mathrm{female}\ \mathrm{and}\ \mathrm{SCR}\le 0.7\ \mathrm{mg}/\mathrm{dl}\\ {}\mathrm{eGFR}=144\times {\left(\mathrm{SCR}/0.7\right)}^{\hbox{-} 1.209}\times {(0.993)}^{\mathrm{age}}& \mathrm{if}\ \mathrm{female}\ \mathrm{and}\ \mathrm{SCR}>0.7\ \mathrm{mg}/\mathrm{dl}\\ {}\mathrm{eGFR}=141\times {\left(\mathrm{SCR}/0.9\right)}^{\hbox{-} 0.411}\times {(0.993)}^{\mathrm{age}}& \mathrm{if}\ \mathrm{male}\ \mathrm{and}\ \mathrm{SCR}\le 0.9\ \mathrm{mg}/\mathrm{dl}\\ {}\mathrm{eGFR}=141\times {\left(\mathrm{SCR}/0.9\right)}^{\hbox{-} 1.209}\times {(0.993)}^{\mathrm{age}}& \mathrm{if}\ \mathrm{male}\ \mathrm{and}\ \mathrm{SCR}>0.9\ \mathrm{mg}/\mathrm{dl}\end{array}} $$

For comparison we also calculated eGFR according to the Modification of Diet in Renal Disease (MDRD) formulae [32]:

$$ {\displaystyle \begin{array}{ll}\mathrm{eGFR}=175\times {\left(\mathrm{SCR}\right)}^{\hbox{-} 1.154}\times {\left(\mathrm{age}\right)}^{\hbox{-} 0.203}\times 0.742& \mathrm{if}\ \mathrm{female}\\ {}\mathrm{eGFR}=175\times {\left(\mathrm{SCR}\right)}^{\hbox{-} 1.154}\times {\left(\mathrm{age}\right)}^{\hbox{-} 0.203}& \mathrm{if}\ \mathrm{male}\end{array}} $$

### Statistical analysis

Since one of the goals was to characterize hyperfiltation, which by definition does not occur in those with established chronic kidney disease, examinations where eGFR was < 60 ml/min/1.73m^2^ were excluded from analysis. Because of the longitudinal nature of these studies, there were often multiple aGFR measurements made in the same participant.

Multiple linear regression models were used to assess associations between independent variables (age, sex, SCR, BSA, diabetes duration, FPG and HbA1c) and the dependent variable, which was aGFR, the inverse of SCR, or the difference between aGFR and CKD-EPI eGFR (defined as aGFR - CKD-EPI eGFR). In these analyses to determine variables associated with aGFR, men and women were analyzed together, and sex was one of the independent variables. The purpose of regressions modelling the inverse of SCR was to find which of the independent variables (excluding the SCR) were most closely associated with creatinine as a clinical measure of GFR. Models were fit using a linear mixed model procedure to account for dependence among multiple measurements in the same individual, with the assumption of an autoregressive correlation structure. Akaike’s Information Criterion (AIC) was used as a measure of goodness of fit for each model; all combinations of variables were tested. The best model has the lowest AIC. These analyses were used to select variables for inclusion in the approximating equations for predicting aGFR. HbA1c and FPG, although highly correlated (*r* = 0.71 in the present data), may have different effects on GFR, thus both were available for selection in the model.

Using the selected variables, an equation for approximating aGFR was developed. First these same data were divided into 8 groups defined by sex and quartiles of SCR. A separate equation was derived for each of the 8 groups; this accounts for nonlinearity over the range of SCR values. (For clinical utility, we did not include diabetes duration in these analyses because it is frequently unknown in patients with type 2 diabetes.) To reduce “overfitting” caused by including a participant’s own data in developing the prediction equation, a 10-fold cross-validation procedure was used. The set of individuals was split into 10 groups of approximately equal size. The models were fit for prediction of aGFR in 90% of the data leaving out one group at a time. The predicted aGFR in each of the 10% of individuals “left out” was calculated from the equation derived in the other 90% of individuals.

To assess fit of the prediction equation, we calculated the correlation coefficient between this predicted GFR and the aGFR. Root mean square error and r^2^ (the proportion of variance in aGFR explained by the model) were also calculated. Differences in predictive properties of different equations were assessed by comparison of the correlation coefficients of each estimate with aGFR; the method of Kleinbaum was used to account for the correlation among the estimates [33]. For purposes of presentation, we show the predicted values for each individual as those derived from the cross-validation procedure, while regression coefficients for the final equations are shown as the average across all 10 “training” sets. The P30 is defined as the probability that an estimated GFR that is within 30% of the aGFR [10].

We also calculated the area under the receiver operating characteristic (ROC) curve for detecting hyperfiltration (defined as aGFR> 120 ml/min/m^2^). This area represents the probability that an equation can distinguish correctly between an individual with hyperfiltration and one without [34]. The difference between the area under ROC curve for the new equation and that for the CKD-EPI equation was tested by the method of DeLong et al. [35]. All statistical analyses were performed with SAS version 9.4 (SAS Institute, Cary, NC).

## Results

**Fig. 1:**
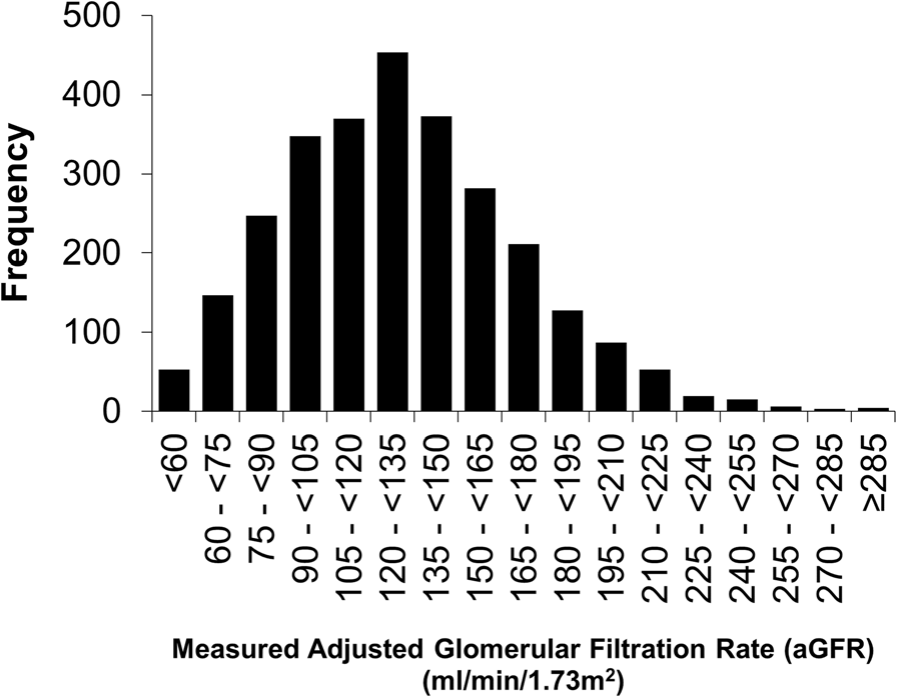
Frequency distribution of measured, aGFRs in 2798 measurements of 269 people in the study cohort over the years 1988–2014

**Fig. 2:**
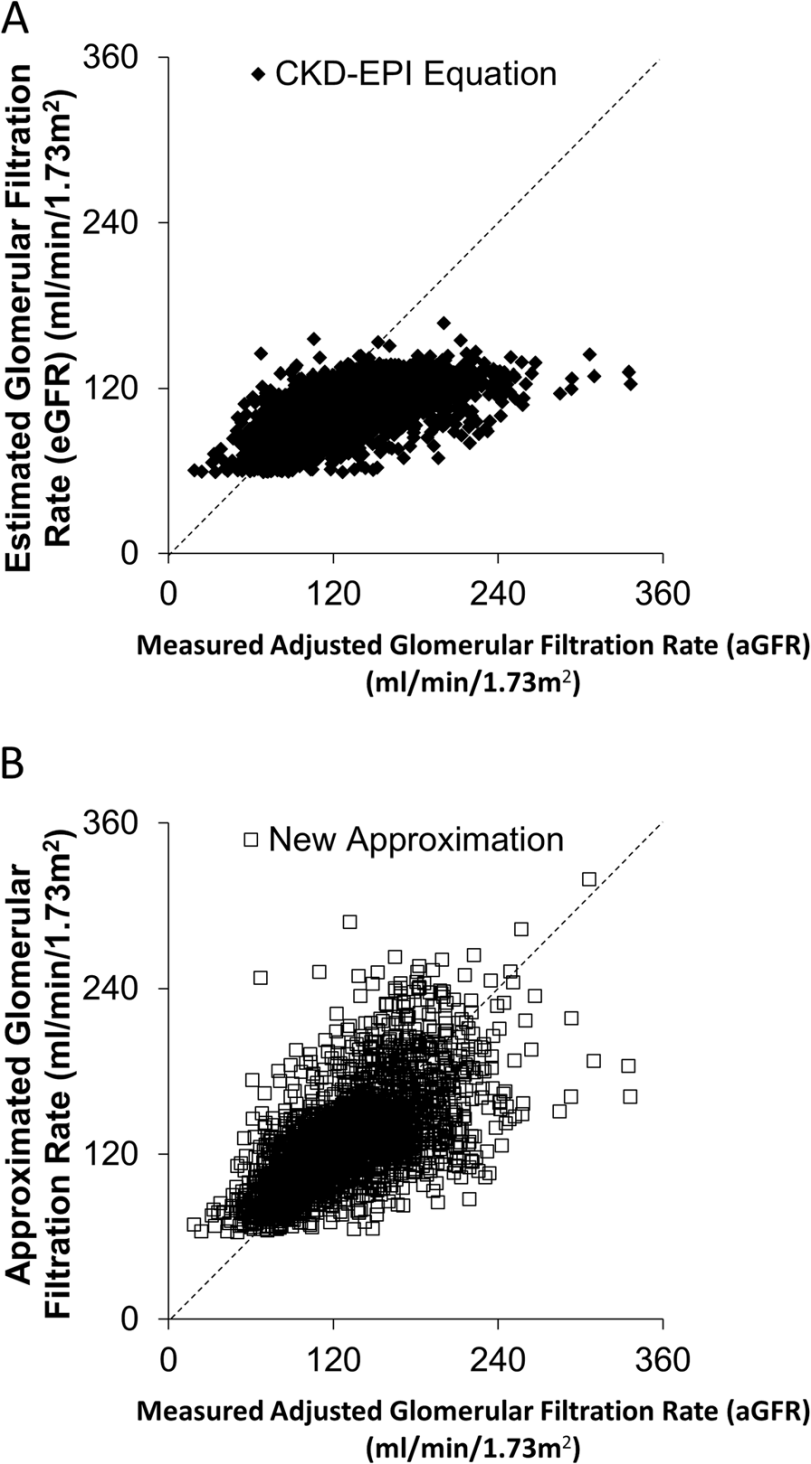
Measured aGFR [(mGFR/BSA) per 1.73m^2^] on the x-axis and the (**a**) CKD-EPI estimated and (**b**) approximated GFR on the y-axis. Black diamonds represent estimations by the CKD-EPI equation and open squares represent approximating equations by new equation. There is better agreement between the measured aGFR and the approximated GFR than there is using the estimating equation CKD-EPI

Glomerular filtration rate was measured at 2798 examinations in 269 individuals where eGFR was greater or equal to 60 ml/min/1.73m^2^. For participants who had aGFR measured more than once, mean time between adjacent aGFR studies was 3.3 ± 1.9 years. Clinical and biological characteristics of participants are summarized in Table 1 and the frequency distribution of measured aGFR is shown in Fig. 1. Figure 2a shows a plot of aGFR versus CKD-EPI eGFR.

**Table 1:** Characteristics of the 269 subjects who participated in 2798 GFR studies over the years 1988–2014

	Men (*N* = 850)		Women (*N* = 1948)	
	Mean	Standard Deviation	Mean	Standard Deviation
Age (years)	49.4	10.0	48.5	11.0
Height (m)	1.718	5.9	1.606	6.0
Weight (kg)	99.3	26.2	94.0	23.4
FPG (mg/dL)	190.9	77.5	214.3	85.7
HbA1c (%)	9.0	2.3	9.7	2.2
Serum creatinine (mg/dL)	0.82	0.17	0.65	0.15
Diabetes duration (years)	12.5	9.5	13.5	8.2
aGFR (ml/min/1.73m^2^)	130.5	39.2	130.4	41.0
CKD-EPI eGFR (ml/min/1.73m^2^)	102.6	16.3	105.7	18.3
BSA (m^2^)	2.10	0.26	1.96	0.22

*FPG* Fasting plasma glucose, *HbA1c* Hemoglobin A1c, *BSA* Body surface area, *aGFR* Adjusted (measured) glomerular filtration rate, *eGFR* Estimated glomerular filtration rate

The best multivariate regression for predicting aGFR, as shown by the smallest AIC, was one that included all variables except BSA; the regression equation is shown in Table 2A. The established variables, age, sex and SCR explained 44.8% of the variance in the logarithm of aGFR, while HbA1c and FPG explained an additional 2.7%. We performed similar analysis to find the best predictors of the inverse of SCR. The inverse of SCR was positively associated with HbA1c as expected, but slightly inversely associated with fasting glucose, as shown in Table 2B. While FPG and HbA1c explained 5.4% of variation in aGFR after adjustment for age and sex, they only explained 1.7% of the reciprocal of SCR. Finally, we used multivariate regression to find which variables best accounted for the difference between CKD-EPI eGFR and aGFR (aGFR−eGFR) (Table 2C). The best model was one that included all the variables except BSA. Although there was a tendency for eGFR from the CKD-EPI equation to underestimate aGFR, high fasting plasma glucose and HbA1c were associated with a larger difference.

**Table 2:** Regression models for aGFR, 1/SCR and aGFR-eGFR

Variable	Β	SE	*P*-value
A	Log aGFR (log ml/min/1.73m^2^)		
Intercept	5.8502	0.0430	
Sex (female vs male)	−0.2151	0.0128	< 0.0001
Age (yrs)	−0.0100	0.0005	< 0.0001
Serum creatinine (mg/dl)	−0.8639	0.0334	< 0.0001
Fasting plasma glucose (10 mg/dl)	0.0061	0.0008	< 0.0001
HbA1c (%)	0.0061	0.0030	0.0402
B	1/SCR (dl/mg)		
Intercept	1.4931	0.0566	
Sex (female vs male)	0.2821	0.0218	< 0.0001
Age (yrs)	−0.0082	0.0009	< 0.0001
HbA1c (%)	0.0287	0.0033	< 0.0001
Fasting plasma glucose (10 mg/dl)	−0.0043	0.0007	< 0.0001
C	aGFR-eGFR (ml/min/1.73m^2^)		
Intercept	39.7124	5.3606	
Sex (female vs male)	−10.5330	1.5884	< 0.0001
Age (yrs)	−0.5928	0.0708	< 0.0001
Serum creatinine (mg/dl)	−11.2485	4.1942	0.0074
Fasting plasma glucose (10 mg/dl)	0.7927	0.0976	< 0.0001
HbA1c (%)	1.0216	0.3789	0.0071
Duration of diabetes	0.1916	0.08687	0.0275

Beta is the regression coefficient and SE is its standard error. Regression models were fit using a mixed model procedure to account for multiple examinations within individuals. Results are shown for best fitting model (lowest AIC)

These same variables were used to develop a prediction equation for aGFR. The final prediction equations are shown in Table 3. The relationship between aGFR and its predicted value using the new approximating equations is shown in Fig. 2b. The correlation between aGFR estimated by the best approximating equations and measured aGFR (iothalamate GFR / BSA) was 0.648 and *r*^2^ = 0.420. The correlation between the aGFR estimated by age and serum creatinine (without glucose variables), on the other hand, was 0.619 and *r*^2^ = 0.383. In comparison, the CKD-EPI equation had a correlation with the aGFR of 0.620 and r^2^ of 0.384; the correlation between aGFR and the new approximating equations was significantly higher than that between aGFR and the CKD-EPI equation (*p* = 1.2 × 10^− 6^ for significance of difference). The correlation between aGFR and an equation derived by 10-fold cross-validation excluding FPG and HbA1c was not significantly different from that between the CKD-EPI equation and aGFR (*p* = 0.63). The MDRD equation had a correlation of 0.554 (*r*^2^ = 0.307) with aGFR. In women, the correlation between aGFR and the new approximating equations was 0.67 (*r*^2^ = 0.44), while that between aGFR and the CKD-EPI equation was 0.63 (*r*^2^ = 0.39, *p* = 8.4 × 10^− 9^ for difference). In men, the corresponding numbers were 0.60 (*r*^2^ = 0.36) and 0.61 (*r*^2^ = 0.37, *p* = 0.52 for difference).

**Table 3:** Intercepts and coefficients of variables in the final approximating equations

		Approximated GFR = e^[x]^ where x =
N	Women	
491	SCR < 0.55 mg/dl	= [4.8623 + (−0.1377 x age) + (0.0290 x HbA1c) + (0.0607 x FPG) + (− 0.0360 x SCR)]
481	0.55 ≤ SCR < 0.62 mg/dl	= [4.8435 + (− 0.1178 x age) + (0.0268 x HbA1c) + (0.0480 x FPG) + (− 0.0039 x SCR)]
492	SCR 0.62 ≤ SCR < 0.72 mg/dl	= [4.7982 + (− 0.1104 x age) + (0.0306 x HbA1c) + (0.0417 x FPG) + (− 0.1263x SCR)]
484	SCR ≥0.72 mg/dl	= [4.7916 + (− 0.0928 x age) + (− 0.0014 x HbA1c) + (0.0671 x FPG) + (− 0.2460 x SCR)]
N	Men	
194	SCR < 0.70 mg/dl	= [4.9756 + (−0.0752 x age) + (0.0357 x HbA1c) + (0.0259 x FPG) + (− 0.1160 x SCR)]
248	0.70 ≤ SCR < 0.79 mg/dl	= [4.9666 + (− 0.1075 x age) + (0.0110 x HbA1c) + (0.0634 x FPG) + (− 0.2750 x SCR)]
224	0.79 ≤ SCR < 0.90 mg/dl	= [4.976 + (− 0.1406 x age) + (− 0.0385 x HbA1c) + (0.0074 x FPG) + (− 0.1463 x SCR)]
214	SCR ≥ -0.90 mg/dl	= [5.0159 + (− 0.1235 x age) + (00.328 x HbA1c) + (− 0.0236 x FPG) + (− 0.1771 x SCR)]

*N* is number of examinations in each group

**Fig. 3:**
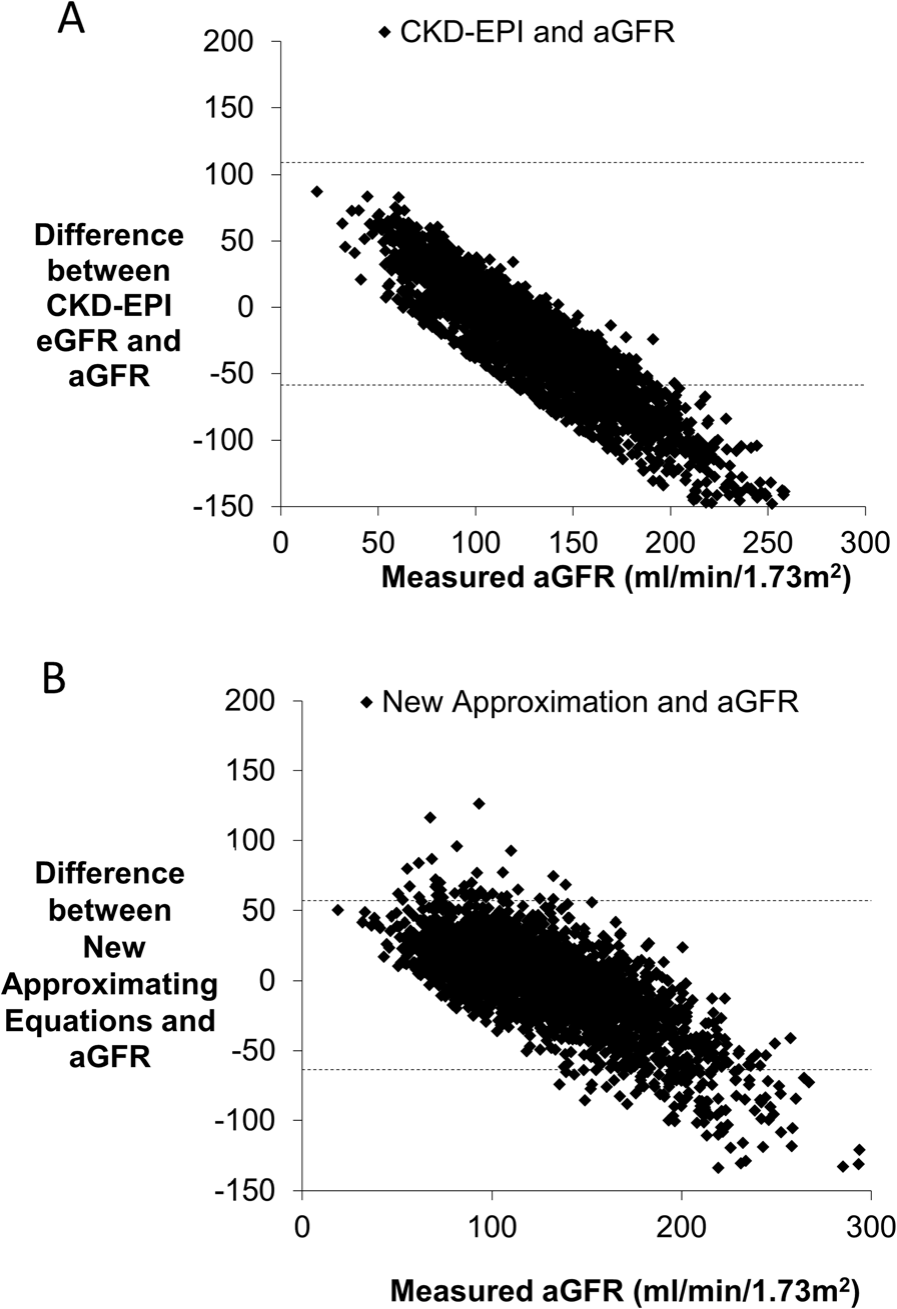
Plots of the (**a**) difference between the CKD-EPI equation and measured aGFR on the y-axis (**b**) difference between the new equation and measured aGFR on the y-axis, both analyzed against the measured aGFR on the x-axis. The CKD-EPI systematically underestimates the aGFR especially at higher values. The new equation distributes error over a wider range of means but also underestimates the aGFR slightly. The mean aGFR was 130.45 ± 40.47 ml/min/1.73m^2^. The mean GFR using the new estimating equations was 126.25 ml/min/1.73m^2^ whereas the mean aGFR using the CKDEPI equation was 104.73 ± 17.75 ml/min/m^2^

Plots of aGFR and the difference between the aGFR and the estimates of the aGFR by each equation show that the CKD-EPI equation overestimates aGFR when it is low and underestimates it when it is high. The mean difference between aGFR and eGFR estimated by the CKD-EPI equation is 25.7 ml/min/1.73m^2^. However, the new approximating equation distributes error over a wider range of means, aGFRs (Fig. 3). The mean difference between aGFR and eGFR by the new approximating equations is 3.3 ml/min/1.73m^2^ (which still underestimates the aGFR on average, but to a much smaller degree). The P30 of the new approximating equation was 82%. By comparison, the CKD-EPI calculated eGFR was within 30% of the aGFR among 75% of measurements.

**Fig. 4:**
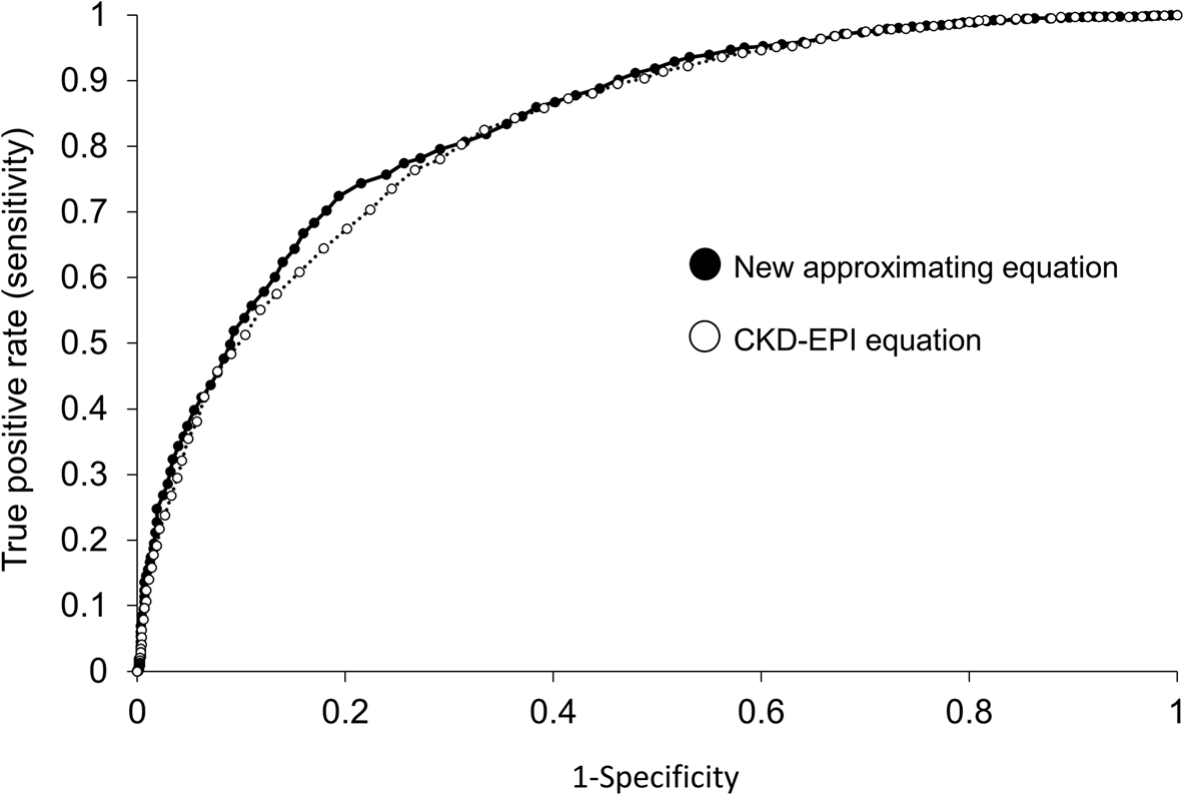
Receiver-operator curve (ROC) showing the false positive rate (1-specificity) on the x-axis and the sensitivity on the y-axis of the new approximating equations versus the CKD-EPI equation

When the new approximating equations were tested against the CKD-EPI equation to find which more accurately estimates hyperfiltration defined as aGFR ≥120 ml/min/1.73m^2^, the area under the ROC curve for the new approximating equation was 0.836 versus 0.825 for the CKD-EPI equation (Fig. 4). This difference was highly statistically significant (*p* = 0.000001). For example, the threshold for detecting hyperfiltration with 80% specificity is ≥109 ml/min/1.73m^2^ by the CKD-EPI equation, and this has sensitivity of 64%. By contrast, the threshold for 80% specificity with the new approximating equations is ≥127 ml/min/1.73m^2^ with a sensitivity of 72%. This represents a statistically significant, but modest, improvement in prediction of hyperfiltration.

## Discussion

In Pimas with T2DM, glycemic variables including FPG, and HbA1c were modestly associated with aGFR, measured by urinary clearance of iothalamate. Thus, the glycemic variables provide additional information about aGFR, along with the usual variables of age, sex and SCR, and these associations can account, in part, for the differences between aGFR and eGFR. The new approximating equations modeled aGFR better than did the CKD-EPI equation, in that they explained significantly more of the variance. Although the additional contribution of glycemia to variance explained is relatively small, it may have clinical importance. However, such clinical relevance has not been demonstrated.

In general, the CKD-EPI eGFR systematically underestimates aGFR and this underestimation is greater in those with hyperglycemia [8–11]. Our analysis was conducted among individuals with eGFR ≥60/ml/min/1.73m^2^ by the CDK-EPI equation, since the CDK-EPI performs well in clinical practice when it is < 60 ml/min/1.73m^2^. This could have biased our results by truncating the range of the CKD-EPI values, but in a sensitivity analysis, elimination of those with aGFR < 60 ml/min/1.73m^2^ gave almost the same results.

The utility of a prediction equation is reflected in accuracy and precision. An equation will generally fit better in the data from which it was derived than in a set of independent individuals. To minimize this “overfitting” bias, we used 10-fold cross-validation. With this approach, there was improvement in the correlation (which largely reflects precision) with the new approximating equations over the CKD-EPI equation, but the correlation of a Pima-specific equation based on the same variables used in CKD-EPI was not significantly different from the CKD-EPI equation. This suggests that the increased precision of the new approximating equations over the CKD-EPI equation is largely due to the use of glycemic information, and not simply due to fitting the model to our own data (and that the 10-fold cross-validation procedure has largely removed any overfitting bias). The correlation of the new equations with the glycemic variables is, of course, strongly significantly higher than the Pima equation without these variables (*p* = 1.9 × 10^− 10^). Inaccuracy of the CDK-EPI equation for predicting aGFR has often been attributed to differences between the populations in which it was derived and those in which it has subsequently been applied [36, 37]. The systematic underestimation of aGFR in the present study is largely due to the population difference. The difference between the aGFR and that by CKD-EPI (which largely reflects accuracy) is 25.7 ml/min/1.73m^2^ higher. This difference is mostly removed when using either of the Pima equations derived from the 10-fold cross-validation procedure. The small difference between the actual and predicted aGFR that we observe with the new equations likely reflects a modest residual nonlinearity in the relationship.

In our analyses SCR was not correlated with glycemic variables to the same extent as was aGFR, so when creatinine-based estimates of GFR are employed in diabetic individuals, information about glycemia-induced hyperfiltration is not captured. Our observation that the difference between eGFR and aGFR is associated with the degree of hyperglycemia is consistent with studies that show that the extent to which eGFR underestimates aGFR depends on the degree of glycemia [38]. Since glycemic variables show more correlation with aGFR than with SCR, it underscores the importance of the non-renal variables used to calculate eGFR. It is possible that the body composition of diabetic people, for example, modifies the relationship between SCR and eGFR, potentially explaining the different directions in correlations of SCR with HbA1c and FPG. However, this study does not include variables such as body mass index, body composition and lipid levels; these might have added to the predictive model. No equation, including the CKD-EPI eGFR, has been validated in a cohort of diabetic subjects with eGFR ≥60 ml/min/m^2^ [38, 39].

We propose that the new approximating equations, which take into account measures of glycemia that the CKD-EPI does not, may be useful when the diabetic patient is NOT approaching end-stage renal disease but might actually be hyperfiltering, i.e. early in diabetes. Using the current reporting system, hyperfiltration might never be uncovered. Using the new approximation of aGFR, the actual value would be reported (rather than “eGFR ≥60 ml/min/1.73m^2^”) so that people with diabetes who are hyperfiltering could be identified and medical decisions made appropriately. One such decision might regard treatment. Newer SGLT2 inhibitors can lower HbA1c and also offer renal protection [40, 41]. Although the Food and Drug Administration does not yet recommend SGLT2 inhibitors for a renal indication, it has been proposed that they may exert renoprotective properties due to the reduction in hyperfiltration [42]. It might be useful to recruit individuals with hyperfiltration into clinical trials to test this hypothesis. If this proves to be the case, it might also be sensible to prescribe SGLT2 inhibitors to those who are hyperfiltering, as one might expect them to have the greatest benefit. The new approximating equations developed here may be useful for identifying such individuals.

Limitations of the present study include the use of the CKD-EPI equation to identify those with low eGFR in order to remove them from the cohort of people with higher aGFRs. Ideally one equation should suffice in the identification of both individuals with low eGFR and those who are hyperfiltering. With the discontinuity in the equation developed here, its optimal use may be in identifying those with hyperfiltration rather than clinical tracking. This equation is actually eight equations, making use complicated though less so with the help of a computer or smart phone. Additionally, many of the assays of creatinine were done before standardization. The effects of drugs like inhibitors of the renin-angiotensin-aldosterone system that can lower the aGFR were not accounted for in this study.

The plots of predicted versus observed aGFR show that the new equation is more accurate than the CKD-EPI equation, but the improvement in terms of variance explained is modest. It is probable that aGFR could be better modeled if there were an endogenous marker of renal function better than creatinine, which has several problems. Not only does it reflect muscle bulk and therefore varies by sex and age, it also shows the effects of hyperglycemia imperfectly, as shown in the present study.

The strengths of this study include the use of a wide range of aGFRs measured by the urinary clearance of iothalamate, division by BSA, and standardization to 1.73m^2^. Another strength of the study is that hyperfiltration was defined at aGFR greater than or equal to 120 ml/min/m^2^ which captures older adults with type 2 diabetes. Since diabetes is the most common cause of kidney failure in the US and, indeed, worldwide, the new equations approximating aGFR represent a potential advance. However, they were developed in a single ethnic group, and applicability to other populations of people with T2DM requires further study and validation.

## Conclusions

In conclusion, aGFRs in Pima people with type 2 diabetes are increased by high FPG and high HbA1c. This information about glycemia-induced hyperfiltration is not reflected in measurements of serum creatinine. These glycemic variables are significant, but of modest effect, in explaining the difference between aGFR and CKD-EPI eGFR. Thus, measures of glycemia are important in approximating aGFR, and possibly for making clinical decisions regarding hyperfiltration.

## Data Availability

The datasets generated and analyzed during the current study are not publicly available due to privacy concerns. Conditional on approval from the Institutional Review Board, the datasets can be made available from the corresponding author upon reasonable request.

## Abbreviations

aGFR: Adjusted glomerular filtration rate
AIC: Akaike’s Information Criterion
BSA: Body surface area
CKD-EPI: Chronic Kidney Disease Epidemiology Collbaroration
DOTA: Tertraxetan
DTPA: Diethylenetriaminpentacetic acid
EDTA: Ethylenediaminetriacetic acid
eGFR: Estimated glomerular filtration rate
FPG: Fasting plasma glucose
GFR: Glomerular filtration rate
HbA1c: Hemoglobin A1c
HPLC: High performance liquid chromatography
P30: Probability estimated GFR is within 30% of aGFR
ROC: Receiver operating characteristic
SCR: Serum creatinine
